# Successful Removal of 265 Cm. of Gangrenous Intestine

**Published:** 1903-04

**Authors:** Roswell Park

**Affiliations:** Buffalo, N. Y., Professor of Surgery, Medical Department, University of Buffalo, 510 Delaware Avenue.


### Successful Removal of 265 cm. of Gangrenous Intestine.

By ROSWELL PARK, M. D., LL. D., Buffalo, N. Y.,
Professor of Surgery, Medical Department, University of Buffalo.

A YOUNG man of 21, previously healthy, living about one hundred miles from Buffalo, was seized with severe pain in the lower part of the abdomen on November 21, 1901. The pain and tenderness were centrally located rather than upon the right side, and I was assured by Dr. Allen, of Olean, that for three days at least there was no perceptible tumor formation. On the third day his temperature ran quite high; two days later it was much lower, and on the morning when he was brought to me his temperature was only 100° F.

Since his first attack of pain and in spite of vigorous catharsis there had been no movement of the bowels. Early in the morning of November 26, *i.e.*, five days after he was taken sick, he was brought to the Buffalo General Hospital, suffering a good deal of pain and requiring considerable anodyne upon the journey. Even at this time his pulse and temperature were lowering, but there was now distinct dulness above the pubes. His tongue and lips were dry and his general condition was indicative of profound sepsis. The abdomen was somewhat distended and rigid. He was occasionally vomiting material of bilious but not fecal character, and there had been no movement of the bowels.

He was promptly operated upon the same day, in clinic, chloroform being the anesthetic used. The incision was made in the middle line. Upon opening the abdomen it was found first that the omentum was firmly bound down, a little more on the right side than on the left, and made to cover in a mass of adherent intestinal coils, from between which as they were gently separated a quantity of very offensive sero-purulent fluid escaped. Some 500 or 600 c.c. of this fluid having been evacuated and the incision being extended from the pubis to the umbilicus, it was made apparent that the primary fault was a gangrenous condition of the appendix, about which three or four coils of small intestine had united themselves and adhered to the cecum, and that all these adherent surfaces were in a gangrenous condition with threatening perforation at numerous points. At least four large areas of this character were found upon the small bowel, the uppermost being apparently near the middle of its length, *i.e.*, a long distance above the cecum. It now became a question whether to undertake several intestinal resections or to remove the entire length of bowel which contained these gangrenous patches. The patient's condition was bad and it seemed that the measure most indicated was that which could be most quickly performed.

Accordingly, I used a Murphy button and with it connected the ascending colon with a loop of intestine just above the uppermost gangrenous area. The mesentery was then tied with a series of silk ligatures, and the entire section of small intestine involved was removed down to the ileocecal valve. A considerable part of the cecum being gangrenous, I divided the colon at this point by an oblique incision, and in this way removed all of its gangrenous portion.

Both ends of the bowel which remained, *i.e.*, small and large, were turned in and sutured, and reliance was placed solely upon the lateral anastomosis made with the button. The abdomen was closed with silkworm gut sutures, a glass drainage tube six inches in length being inserted, its lower end being deep in the pelvis. Infusion of salt solution was made during the operation and at one time it seemed as though the patient could not be gotten off the table alive.

After the operation the portion of intestine which was removed was straightened out and carefully measured. Without undue stretching it was found to measure 265 cm.=8 ft. 9 in.

During the first 24 hours after the operation the patient had intense pain requiring 30 cg. of morphine for his relief; but after this he became much more comfortable. From 3 to 6 cc. of fluid were drained off every two or three hours. Forty-eight hours later this fluid began to have a fecal odor. During this time his bowels had moved repeatedly. In fact, he suffered from such looseness and incontinence of feces as to require opium and starch water injections into the rectum. By the end of the second day his temperature was below 100° F. and his pulse was 120; he suffered from restlessness rather than from pain. On the fifth day the discharge through the drainage tube assumed a distinctly fecal character. A rubber tube was substituted for the glass.

The patient had a distinct fecal fistula for 3 or 4 months, although the amount discharged during the latter part of the time was very slight. All this time his bowels moved in perfectly natural manner and his appetite was more than normal. So far as could be, his diet was limited to that which the stomach and upper intestine could best digest.

The Murphy button did not come away until the 10th of March following, nearly 4 months after its insertion. After its passage there was a notable improvement in the fistulous condition. This eventually closed perfectly and the patient completely regained his health and strength. Eleven months later he reported by letter that he weighed as much as he ever did, enjoyed good appetite, and felt himself in the best of health in every respect.

This was the most extensive case of gangrene of intestines, following a primary gangrenous appendicitis, that has ever come to my notice. The history of events had evidently been as follows: primary affection of the appendix with rapid gangrene, localised peritonitis with agglutination of the neighboring coils of small intestine, rapid infection of all the surfaces thus brought in contact with the primary focus, and prompt necrosis due in all probability to the virulence of the infecting organisms.

Some years ago I began to maintain that most cases of general peritonitis, which were not plainly due to some ordinarily easily recognised lesion, took their real origin from an inflamed appendix. In other words,—that is, to put it a little differently,—the appendix is at fault in most cases of so-called spontaneous peritonitis, and the right iliac fossa should be carefully interrogated in all cases of this general character. Furthermore, in many of these cases it would be wise to operate unless the patient be already moribund. I would agree with Dr. Joseph Price, that when there are symptoms of obstruction of the bowel we should open in the middle line; when the bowels are not obstructed the opening may be made over the cecum.

The relation of primary appendicitis to general peritonitis is now very much more widely appreciated than it was a few years ago, and yet there is need of insistence upon this possibility.

The location of the pain as described by the patient is not always a safe but is often a misleading sign. In this particular instance the patient's first complaint of pain was held to point rather to the general peritoneal cavity than to the appendix. I have repeatedly seen cases that had proceeded to gangrene where the pain was referred to the left side rather than to the right. In these cases there seems sometimes to occur even a transfer of tenderness, although this latter indication much less frequently misleads. Muscle spasm begins nearly always on the right side, but in severe cases will involve the entire abdominal musculature. As the infectious processes and ensuing necrosis extend, there will often ensue a reduction of temperature, even to subnormal, and a perceptible reduction of pulse rate. Occurring in this way these indications are not favorable but are rather to be dreaded. Chill does not always occur even though pus accumulates in considerable quantities. It is of the gravest import should it happen, but many of these cases may die without it.

I have gathered from literature sixteen cases, beside my own, where more than 200 cm. of bowel have been removed. Cases of removal of shorter sections are now so numerous that they have, perhaps, lost their principal surgical interest.

It is of interest that Blayney, reporting a case of this kind for Hayes in the *British Medical Journal* for November 16, 1901, took occasion to say that it seemed to him that to remove more than 200 cm. of intestine was to approach, if not to exceed, the danger line regarding future capacity for absorption of the necessary nutrition.

Obalinski's case, the last in the subjoined table, was one of removal of 365 cm. of intestine, but this happened to be in a boy, and comprised nearly his entire intestinal tract. This case promptly died. The other fatal cases, *i.e.*, Maydl's and Von Eiselsberg's, lived long enough to recover from the actual shock of operation and were the only ones who died. The other fifteen cases all recovered although from 205 to 330 cm. were exsected.

CASES OF INTESTINAL RESECTION WITH REMOVAL OF MORE THAN 200 CM. OF INTESTINE.

| | Operator. | Amount Removed. | Result. |
|---|---|---|---|
| 1 | Koeberle . . . . . | 6 feet 10 inches = 205 cm. . | . Recovered. |
| 2 | Kocher . . . . . | 6 " 11 " = 208 " . . . | " |
| 3 | Dressman . . . . . | 7 " 2 " = 215 " . . . | " |
| 4 | Shepherd . . . . . | 7 " 9 " = 234 " . . . | " |
| 5 | Kukula . . . . . | 7 " 9 " = 237 " . . . | " |
| 6 | Harris . . . . . | 7 " 10 " = 239 " . . . | " |
| 7 | Hayes . . . . . | 8 " 4½ " = 248 " . . . | " |
| 8 | Peck . . . . . . . | 8 " 5½ " = 251 " . . . | " |
| 9 | Lauwers . . . . . . | 8 " 9 " = 265 " . . . | " |
| 10 | Park, R. . . . . . | 8 " 9 " = 265 " . . . | " |
| 11 | Payr . . . . . . . | 9 " ½ " = 275 " . . . | " |
| 12 | Maydl . . . . . . | 9 " 4 " = 284 " . . | Died 3 weeks later of inanition. |
| 13 | Fantino . . . . . | 10 " 4 " = 310 " . . . | Recovered. |
| 14 | Monprofit . . . . | 10 " 4 " = 310 " . . . | " |
| 15 | Ruggi . . . . . . | 11 " " = 330 " . . . | " |
| 16 | Von Eiselsberg . . . | 11 " 8 " = 350 " . . . | Death after 25 d'ys. |
| 17 | Obalinski . . . . . | 12 " 2 " = 365 " . . . | Died. |

In cases 9, 11, 14 and 17, from 8 to 30 cm. of large intestine were included in the measurements given.

This table was largely compiled from the tables of Payr, (*Archiv. f. Klin. Chir.*, Bd. 67, S 193,) and Harris (*New York Medical Record*, October 11, 1902, p. 566.)

It is interesting to compare these results, after removal of over 200 c.m. with twenty-five cases (taken from Harris's table) where from 80 to 196 cm. were removed. Of these but 16 recovered, while 9 died within a week after the operation.

510 DELAWARE AVENUE.

